# Structural exploration with AlphaFold2-generated STAT3α structure reveals selective elements in STAT3α-GRIM-19 interactions involved in negative regulation

**DOI:** 10.1038/s41598-021-01436-7

**Published:** 2021-11-30

**Authors:** Seema Mishra, Santosh Kumar, Kesaban Sankar Roy Choudhuri, Imliyangla Longkumer, Praveena Koyyada, Euphinia Tiberius Kharsyiemiong

**Affiliations:** Department of Biochemistry, School of Life Sciences, University of Hyderabad, Hyderabad, 500046 India

**Keywords:** Computational biology and bioinformatics, Structural biology

## Abstract

STAT3, an important transcription factor constitutively activated in cancers, is bound specifically by GRIM-19 and this interaction inhibits STAT3-dependent gene expression. GRIM-19 is therefore, considered as an inhibitor of STAT3 and may be an effective anti-cancer therapeutic target. While STAT3 exists in a dimeric form in the cytoplasm and nucleus, it is mostly present in a monomeric form in the mitochondria. Although GRIM-19-binding domains of STAT3 have been identified in independent experiments, yet the identified domains are not the same, and hence, discrepancies exist. Human STAT3-GRIM-19 complex has not been crystallised yet. Dictated by fundamental biophysical principles, the binding region, interactions and effects of hotspot mutations can provide us a clue to the negative regulatory mechanisms of GRIM-19. Prompted by the very nature of STAT3 being a challenging molecule, and to understand the structural basis of binding and interactions in STAT3α-GRIM-19 complex, we performed homology modelling and *ab-initio* modelling with evolutionary information using I-TASSER and *avant-garde* AlphaFold2, respectively, to generate monomeric, and subsequently, dimeric STAT3α structures. The dimeric form of STAT3α structure was observed to potentially exist in an anti-parallel orientation of monomers. We demonstrate that during the interactions with both unphosphorylated and phosphorylated STAT3α, the NTD of GRIM-19 binds most strongly to the NTD of STAT3α, in direct contrast to the earlier works. Key arginine residues at positions 57, 58 and 68 of GRIM-19 are mainly involved in the hydrogen-bonded interactions. An intriguing feature of these arginine residues is that these display a consistent interaction pattern across unphosphorylated and phosphorylated monomers as well as unphosphorylated dimers in STAT3α-GRIM-19 complexes. MD studies verified the stability of these complexes. Analysing the binding affinity and stability through free energy changes upon mutation, we found GRIM-19 mutations Y33P and Q61L and among GRIM-19 arginines, R68P and R57M, to be one of the top-most major and minor disruptors of binding, respectively. The proportionate increase in average change in binding affinity upon mutation was inclined more towards GRIM-19 mutants, leading to the surmise that GRIM-19 may play a greater role in the complex formation. These studies propound a novel structural perspective of STAT3α-GRIM-19 binding and inhibitory mechanisms in both the monomeric and dimeric forms of STAT3α as compared to that observed from the earlier experiments, these experimental observations being inconsistent among each other.

## Introduction

**... the structure of every organic being is related, in the most essential yet often hidden manner, to that of all the other organic beings** **...**

**—Charles Darwin, The Origin of Species.**

Human Signal Transducer And Activator Of Transcription 3 (STAT3), a key transcription factor, performs a dual role in cancers, it can act as an oncogene or a tumor suppressor depending upon the pathways in which it is involved (1). The transcriptional regulatory activities of STAT3 are mediated by a plethora of post-translational modifications (PTMs) that involve phosphorylation, acetylation and methylation of specific residues as well as dimerization (2–3). PTMs enable it to play diverse functional roles in a host of metabolic, developmental and anti-inflammatory processes. A multi-domain protein, it is known to shuttle between the cytoplasm, nucleus and mitochondria. In the cytoplasm, it exists as a dimeric molecule, with dimerization being enabled by Tyr705 phosphorylation. This protein is then translocated to the nucleus where it can bind to respective response element in the DNA; while in the mitochondria, it exists mostly in a monomeric form. Both the nuclear and mitochondrial localization sequences have been found adorning the STAT3 sequence.

The full-length STAT3 protein is composed of multiple domains. These domains are categorized as follows: 1–130: N-terminal domain (NTD); 130–320: coiled coil domain (CC); 321–465: DNA-binding domain (DBD); 466–585:linker domain (LD); 586–688:Src homology 2 domain (SH2); 689–722:pY and 723–770: transactivation domain (TAD) (1). It is present as two major isoforms, STAT3α and STAT3β, with C-terminal residue changes and truncation present in the latter isoform. We have used the full-length isoform STAT3α throughout our studies.

The NTD of STAT3 mediates two important processes: weaker DNA binding site’s recognition and anti-parallel dimer formation of unphosphorylated form of STAT3 (4). CC participates in the nuclear translocation and in IL-22R signalling activities. While DBD binds directly to the DNA, LD domain that occurs between DBD and SH2 domain may play a structural role as an allosteric communicator between these two (5). SH2 domain is involved in the main dimer formation and pY region is required for the phosphorylation of Y705, which activates STAT3 to carry out its function of regulation of gene expression of several genes involved in cell differentiation and cell proliferation processes. In addition to the activation via Y705 phosphorylation by cytokines, STAT3 is activated by S727 phosphorylation by MAPKs. This residue is present in the TAD region, an intrinsically disordered region located at the C-terminal end.

Besides phosphorylated STAT3, unphosphorylated STAT3 can also localize to the nucleus, form dimers and bind to the DNA. Unphosphorylated STAT3, in its capacity as a dimer, influences several activities associated with an activated transcription factor: nuclear localization, DNA-binding, chromatin-remodeling and specific gene expression regulation (6, 7). Phosphorylation is not the only post-translational mechanism occurring in STAT3, acetylation and methylation are also one of the many possible mechanisms involved (2, 3). These may or may not affect DNA-binding and other activities. As an example, Belo et al. (8) found that Lys685 acetylation alone had no effect on the crystal structure of Tyr705-phosphorylated STAT3 in complex with DNA, which was found identical to the crystal structure of Y705-phosphorylated STAT3, and so, the DNA-binding activity is unaffected.

Gene associated with Retinoid–IFN-induced Mortality-19 protein, abbreviated as GRIM-19, which associates with STAT3 to modulate its activity, was initially identified as an interferon (IFN)-β and retinoic acid (RA)-inducible gene (9). Its overexpression was found to enhance cell death in response to IFN-β/RA. It was found localized to the cytoplasm and nucleus and subsequently as a component of mitochondrial NADH:ubiquinone oxidoreductase (complex I) (10).

GRIM-19 was found associated with STAT3 as observed from a yeast 2-hybrid assay (11, 12), and to negatively regulate STAT3 activity. On the basis of its specific interactions with STAT3 and negligible interactions with STAT1, STAT2 and STAT5 (11), it was postulated to be an effective anti-oncogenic and anti-inflammatory agent, binding to STAT3 and suppressing its activity.

Lufei et al. (12) found that while coiled-coil, DNA-binding and linker domains of murine STAT3 interacted with GRIM-19, there was no association of the N-terminal domain, the SH2 domain or the C-terminal domain. Specific regions of GRIM-19 in interaction with STAT3 were mapped to the region comprising specifically of 36–72 amino acid residues in the region 36–101, which harbors the interacting region. While this study used murine constructs, there is a dearth of such information on human STAT3 protein. Human STAT3 (UniProt ID P40763) and human GRIM-19 (NCBI ID NP_057049.5) are 99% and 83% identical to murine STAT3 (UniProt P42227) and GRIM-19 (UniProt ID Q9ERS2), respectively. However, Zhang et al. (11), found only the transactivation domain of STAT3 to be bound by GRIM-19. While GRIM-19 is found localized to the cytoplasm, its N-terminal domain is also found to harbour the mitochondrial and nuclear localization sequence (12). In the mitochondria, STAT3 associates with GRIM-19, and is imported (11, 13).

As noted from above, there are discrepancies in the nature and location of exact binding sites of STAT3α and GRIM-19. Here, we report the specific interaction sites in GRIM-19 and unphosphorylated and phosphorylated STAT3α, in both the monomeric and dimeric forms. We also investigate the interfacial mutations involved in the changes in binding affinities and stability, and pinpoint key residue mutations playing a role in complex destabilization. Elucidation of the structural basis of STAT3α-GRIM-19 binding, interactions and mutations will lead towards a greater understanding of GRIM-19 as an effective anti-oncogenic therapeutic target.

## Results

### STAT3 structure: I-TASSER

**Figure 1:**
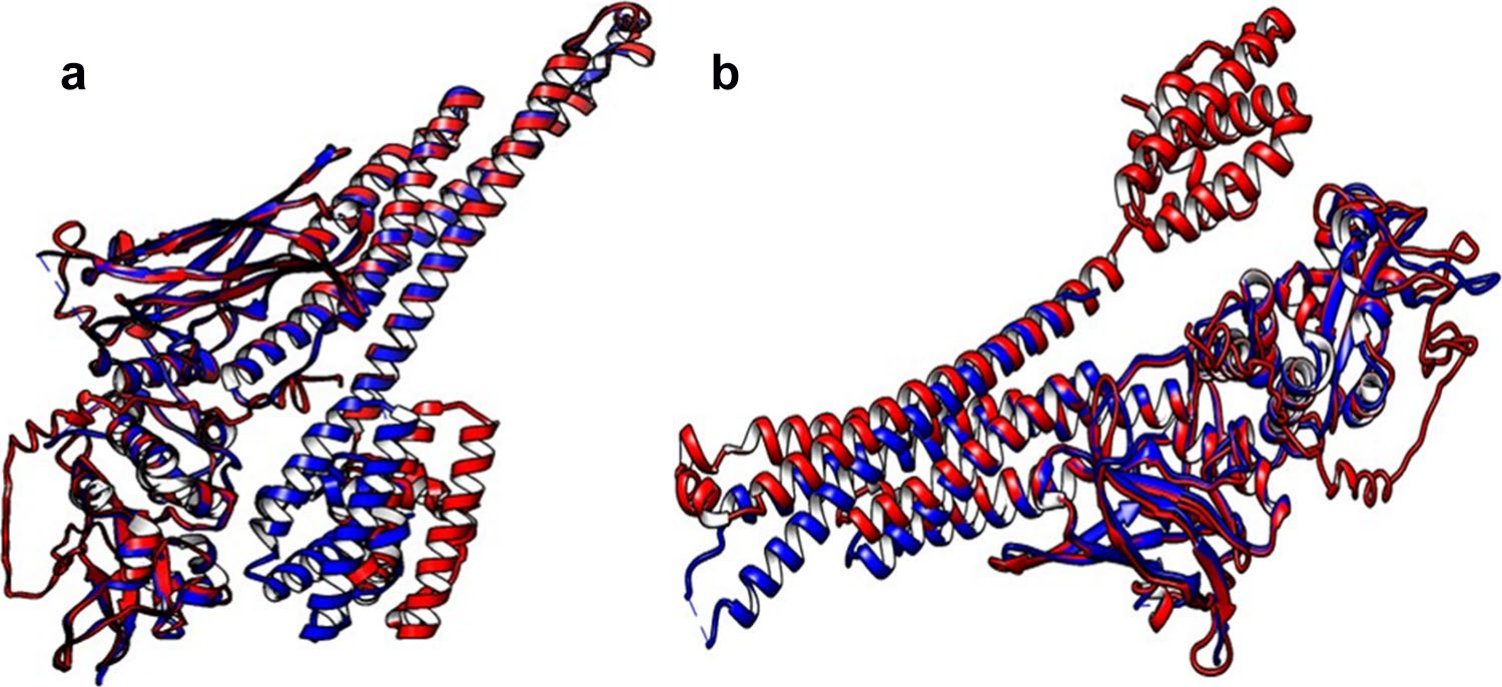
(**a**) I-TASSER-generated human STAT3α structure (in blue) modeled and superimposed on human STAT1 (PDB ID: 1YVL, in red) (**b**) I-TASSER-generated human STAT3α full-length structure (in red) with NTD included, superimposed on STAT3 (in blue) with PDB ID: 6TLC, which does not include NTD.

Due to the lack of absolute clarity on the exact binding sites or domains of STAT3 to which GRIM-19 binds, we wanted to identify the involved domains in the multi-domain STAT3 structure and GRIM-19. We further attempted to understand the structural basis of human GRIM19 and human STAT3 binding through studies on protein–protein complex formation and consequent interactions. Using powerful computational modeling, prior findings can be recapitulated or newer hypotheses can be generated. Towards this end, we wanted to determine the mechanisms of protein–protein association in the case of GRIM-19 with monomeric STAT3, which is usually present in the mitochondria and with dimeric STAT3, usually present in the cytoplasm. We scouted for their respective crystal structures in the PDB. While a human GRIM19 crystal structure with full sequence is readily available (5XTD, sequence 100% identical to NCBI RefSeq, Reference Sequence: NP_057049.5, 144 aa residues), that of unphosphorylated, full-length human STAT3α (770 aa residues) is not crystallized yet. A couple of PDB structures of human STAT3 (6TLC, 598 aa and 6QHD, Y705-phosphorylated, 596 aa residues) have appeared recently in the PDB, but could not be taken for our studies as these are not full-length structures. Further, we did not proceed with the murine STAT3 structure (3CWG) present in PDB, first, for want of a full sequence and structure and second, because this is a well-known fact that the sequence identity does not always translate into structural similarity, and *vice-versa,* with hemoglobin and myoglobin proteins being prime examples. Therefore, in order to obtain a complete structure, we subjected STAT3 sequence taken from UniProt ID P40763 (corresponding to NCBI Reference Sequence: NP_644805.1) to homology modeling with 3D structure prediction tools, I-TASSER and SwissModel. Both these well-validated tools consistently identified, with high confidence, human STAT1 with PDB ID 1YVL B chain, as a significant template on which human STAT3α was modeled (Fig. 1a). This 1YVL accession ID refers to an unphosphorylated form of human STAT1 molecule. BLASTp pairwise sequence alignment produced 54.44% sequence identity between human STAT1 and human STAT3α molecules. Furthermore, both these tools did not identify the murine STAT3 (PDB ID 3CWG) as a significant template. Superimposition of our full-length modeled structure on the partial STAT3 crystal structure (Fig. 1b) showed the differential orientation of coiled coil region due to the inclusion of NTD in our structure.

### STAT3 structure: AlphaFold2

**Figure 2:**
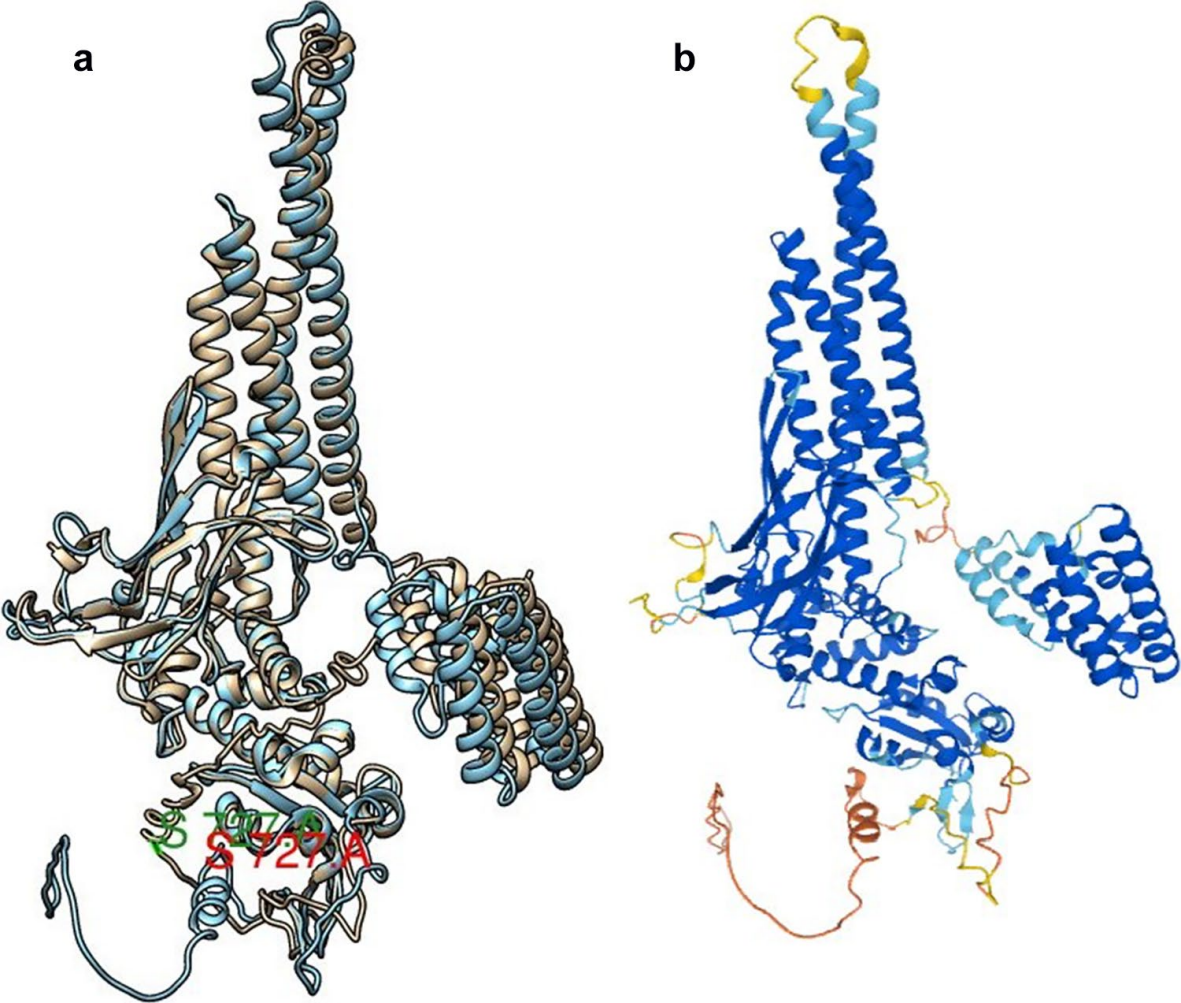
(**a**) Superimposed structures of STAT3, I-TASSER-generated structure is in gold color while AlphaFold2-generated one is in blue color. Ser727 residue is labelled in each structure. The NTD region, a part of CC region and TAD region is seen displaced with respect to each other. (**b**) AlphaFold2-derived structure: model confidence as per pLDDT values, colored in blue: very high confidence regions, pLDDT > 50, colored in orange and yellow: very low confidence regions, pLDDT < 50.

While we were working with our homology-modeled structure, AlphaFold2 predicted structures were hosted at the EBI database. Therefore, in order to carry out comparison studies, we also utilized this newly minted neural-network based structure prediction tool, which is believed to provide an atomic-level accuracy of the protein structures. Towards this end, we downloaded the PDB file of human STAT3 corresponding to the same UniProt ID P40763 from AlphaFold2 protein structure database hosted at EBI. The superimposition of I-TASSER- and AlphaFold2-generated structures was done by MatchMaker in ChimeraX. Across all the 770 atom pairs, RMSD between 501 pruned atom pairs was 0.665 angstroms (Fig. 2a), which indicates similar atomic positions in these two structures. The NTD and C-terminal regions are far less superimposed than the middle regions of the structures. The divergence between other atoms may most often be due to the long disordered region at the C-terminal TAD. This C-terminal region is shown with a low degree of model confidence in AlphaFold2 (pLDDT < 50), while the majority of the structure enjoys a high degree of confidence (very high (pLDDT > 90)), with few exceptions scattered across (Fig. 2b).

#### STAT3 structure: model quality assessments

We used the first generated model with the highest c-score. According to I-TASSER developers, “C-score is a confidence score for estimating the quality of predicted models by I-TASSER. It is calculated based on the significance of threading template alignments and the convergence parameters of the structure assembly simulations”.

The minimized STAT3 model quality was also assessed by multiple model quality assessment tools. For I-TASSER generated model, ERRAT calculated the overall quality factor as 89.239%, very near the 91% cutoff, while VERIFY3D assessed 83.90% of the residues to have averaged 3D-1D score >  = 0.2. PROCHECK (PDBsum) statistics found 77%, 19.1%, 2.9% residues in the most favored, additionally allowed and generously allowed regions, respectively (Table S1 in supplementary material).

For AlphaFold2 generated model, the model quality assessment was as follows: ERRAT overall quality factor score: 94.95%, while VERIFY 3D assessment failed with 77.92% of the residues having averaged 3D-1D score >  = 0.2. PROCHECK hosted at PDBsum (EBI) statistics were as follows: 90.5%, 8.3% and 0.9% residues in the most favored, additionally allowed and generously allowed regions, respectively (supplementary materials S1).

Unphosphorylated STAT3 was then subjected to the addition of a phosphoryl group at the S727 residue, in order to generate a phosphorylated form. S727 is found to be required for the transactivation activity of TAD domain which is bound by GRIM-19 (11). We then subjected both unphosphorylated and phosphorylated human STAT3 and human GRIM19, after minimization, to blind docking using ClusPro2. ClusPro2 has consistently ranked among the top-most protein–protein docking tools in CAPRI assessments (https://abcgroup.ClusPro2.org/2020/01/16/ClusPro2-ranks-first-in-7th-capri-evaluation-round/) and therefore, the protein–protein complex models generated using ClusPro2 can be taken with a high degree of confidence.

### STAT3-GRIM-19 binding and interactions

**It has been recognized that hydrogen bonds restrain protein molecules to their native configurations, and I believe that as the methods of structural chemistry are further applied to physiological problems it will be found that the significance of the hydrogen bond for physiology is greater than that of any other single structural feature.**

**— Linus Pauling**

**Nature of the Chemical Bond and the Structure of Molecules and Crystals (1939), 265.**

#### Full-length unphosphorylated and phosphorylated monomeric STAT3 in interactions with GRIM-19: I-TASSER Model

**Figure 3:**
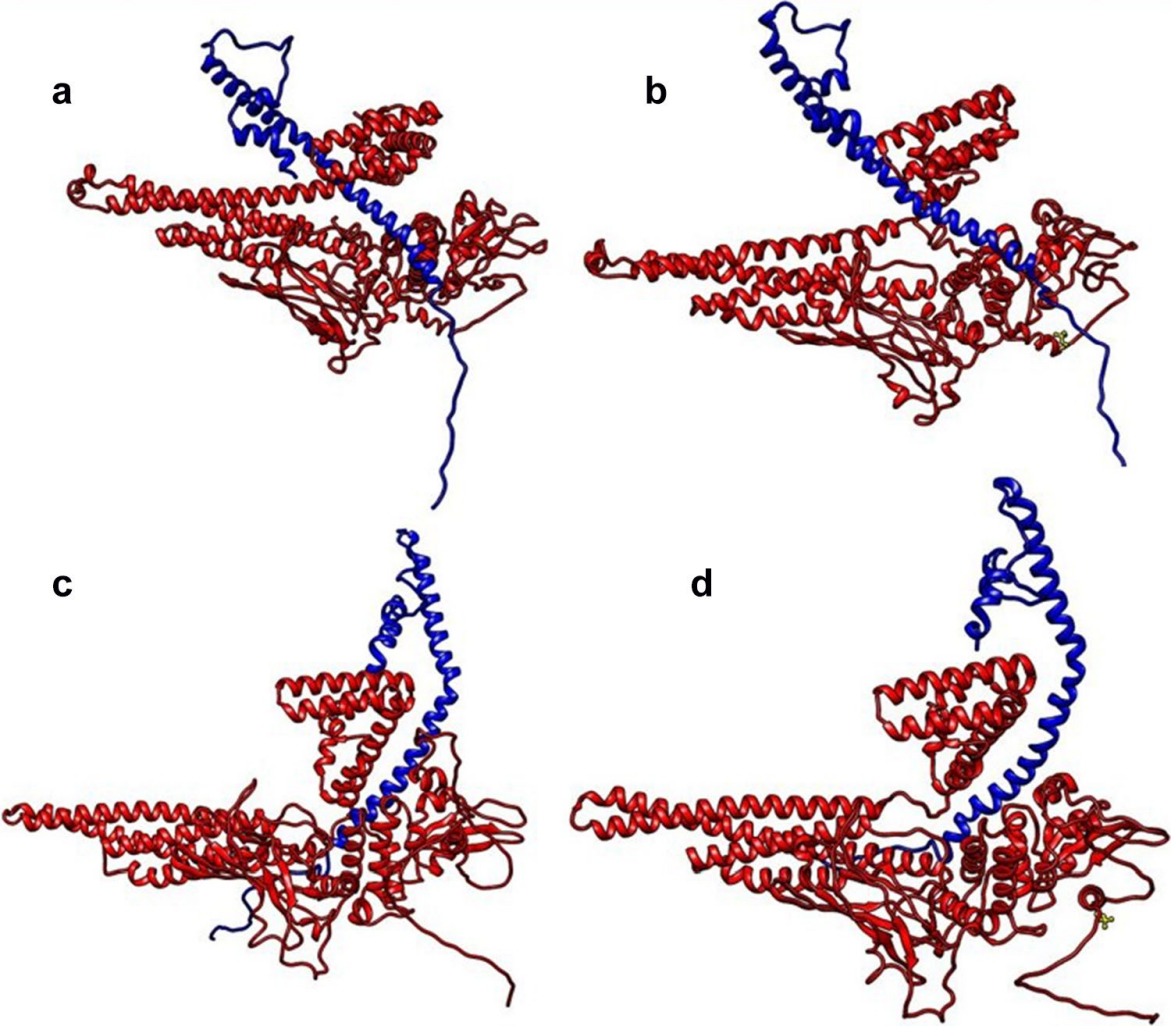
ClusPro2 docked complexes, (**a**) and (**b**) I-TASSER-generated STAT3; unphosphorylated and phosphorylated STAT3-GRIM-19, respectively (**c**) and (**d**) AlphaFold2-generated STAT3; unphosphorylated and phosphorylated STAT3-GRIM-19, respectively. STAT3 in red and GRIM-19 in blue, phosphoryl group on Ser727 is seen as ball and stick representation in yellow in b and d panels.

We first performed blind docking of the two proteins, full-length STAT3 and GRIM-19, where residues were not specified to be involved in the interactions, using ClusPro2, a well-validated protein–protein docking tool which is widely used (14, 15). Using I-TASSER-generated structure for complex formation, among a total of 22 hydrogen-bonded interactions for unphosphorylated STAT3, we found that the major regions involved were mostly from the NTD region (Fig. 3a), and a couple of residues from LD, SH2 and pY domains in the case of STAT3, and specific residues between positions 33 to 79 in the NTD of GRIM19 (Table 1). Some residues located in the NTD of GRIM19 were in multiple hydrogen-bonded interactions with several residues of STAT3. This is interesting in view of the fact that the NTD of GRIM-19 as well as its C-terminal region has been shown to be important for maintaining the transmembrane potential of the mitochondria (16). STAT3 protein, phosphorylated on S727 residue, displayed 29 hydrogen bonds with GRIM-19 (Fig. 3b and Table 1). The complexes were then scrutinized for those complexes harbouring S727 in direct hydrogen bond interactions with GRIM-19 residues. We found that one of the complexes with phosphorylated STAT3, ranked 6th according to ClusPro2 cluster size, harboured phosphorylated Ser727 in hydrogen bonding interactions with Arg115 residue of GRIM19, but the unphosphorylated Ser727 did not interact via hydrogen bonds.

**Table 1:** I-TASSER-generated STAT3 structure: Hydrogen bond interactions between STAT3 and GRIM-19 complexes generated by ClusPro2.

Unphosphorylated STAT3 (A)-GRIM-19 (W)	Phosphorylated STAT3 (A)-GRIM-19 (W)
W:ARG27:HH11—A:ASP698:OD2	W:ARG27:HH11—A:ASP698:OD2
W:ARG55:HH21—A:GLU16:OE1	W:ARG55:HH21—A:GLU16:OE1
W:ARG58:HH11—A:GLU29:OE1	W:ARG58:HH11—A:GLU29:OE1
W:ARG68:HH11—A:GLU74:OE2	W:ARG68:HH11—A:GLU74:OE2
A:ASN5:HD22—W:GLU66:OE2	A:ASN5:HD21—W:GLU66:OE2
A:GLN32:HE22—W:GLN61:O	A:GLN8:HE21—W:GLU66:OE2
A:ASN76:HD22—W:ASP64:OD2	A:SER23:HG—W:MET51:SD
A:TYR79:HH—W:ASP64:OD2	A:GLN32:HE21—W:GLN61:O
A:LYS517:HZ3—W:TYR45:OH	A:ASN76:HD21—W:ASP64:OD2
A:ASN646:HD22—W:SER31:O	A:TYR79:HH—W:ASP64:OD2
W:ARG27:HH11—A:ASP698:O	A:LYS517:HZ3—W:TYR45:OH
W:ARG27:HH21—A:ASP698:O	A:ALA578:H—W:GLY41:O
W:TYR33:H—A:ASN646:OD1	A:ASN646:HD21—W:SER31:O
W:TYR33:HH—A:GLU696:OE1	A:SER649:HG—W:TYR33:OH
W:SER34:H—A:ASN646:OD1	W:ARG27:HH21—A:ASP698:O
W:SER34:HG—A:ASN646:OD1	W:ARG27:HH22—A:ASP698:O
W:TYR45:HH—A:SER514:O	W:TYR33:H—A:ASN646:O
W:ARG57:HH11—A:CYS765:SG	W:TYR33:HH—A:GLU652:OE1
W:ARG57:HH21—A:CYS765:SG	W:SER34:H—A:ASN646:OD1
W:ARG58:HH12—A:GLN32:OE1	W:SER34:HG—A:ASN646:OD1
W:GLN61:HE22—A:GLU29:OE2	W:TYR45:HH—A:SER514:O
W:ARG58:CD—A:GLN32:OE1	W:TYR45:HH—A:THR515:O
	W:TRP53:HE1—A:CYS765:O
	W:ARG57:HH11—A:CYS765:SG
	W:ARG57:HH21—A:CYS765:SG
	W:ARG58:HH12—A:GLN32:OE1
	W:GLN61:HE22—A:GLU29:OE2
	A:ARG688:CD—W:TYR33:OH
	W:ARG58:CD—A:GLN32:OE1

#### Full-length unphosphorylated and phosphorylated monomeric STAT3 in interactions with GRIM-19: AlphaFold2 Model

The same blind docking method as above was used for AlphaFold2-generated STAT3 structure. In this case, STAT3 (unphosphorylated) in complex with GRIM-19 (Fig. 3c), exhibited 38 hydrogen bonds (Table 2). Again, the major STAT3 residues involved in the hydrogen bonding were mostly from NTD, as well as a few residues from LD, SH2 and CC domains. The S727 phosphorylated form of STAT3-GRIM-19 complex (Fig. 3d) showed 39 hydrogen bonds between them (Table 2). The complexes harbouring direct hydrogen bonding interactions of Ser727 were ranked 7th in unphosphorylated and 4th in phosphorylated forms of STAT3, according to ClusPro2 cluster size. The GRIM-19 residues involved in these latter interactions with S727 were Glu66 and Arg68 for the unphosphorylated one and Arg57, Arg58 and Gln61 for the phosphorylated complex (Table 2).

**Table 2:** AlphaFold2-generated STAT3 structure: Hydrogen bond interactions between STAT3 and GRIM-19 complexes generated by ClusPro2.

Unphosphorylated STAT3 (A)-GRIM-19 (W)	Phosphorylated STAT3 (A)-GRIM-19 (W)
A:ARG103:HH11—W:GLU66:OE1	A:ARG103:HH11—W:GLU66:OE1
A:ARG103:HH21—W:GLU66:OE2	W:ARG23:HH21—A:GLU238:OE1
W:ARG27:HH11—A:ASP242:OD1	W:ARG27:HH11—A:GLU238:OE2
W:ARG55:HH22—A:GLU582:OE2	W:ARG55:HH22—A:GLU582:OE2
W:ARG57:HH12—A:GLU111:OE1	W:ARG57:HH21—A:GLU111:OE1
W:ARG68:HH11—A:GLU50:OE1	W:ARG59:HH12—A:GLU681:OE2
A:ARG13:HH12—W:THR42:OG1	W:ARG68:HH11—A:GLU50:OE1
A:GLN17:HE22—W:THR42:O	A:ARG13:HH12—W:THR42:OG1
A:ARG103:HH12—W:GLN61:O	A:GLN20:HE21—W:HIS47:NE2
A:ARG107:HH12—W:ASN54:O	A:ARG103:HE—W:GLN61:O
A:ARG114:HH11—W:ASN54:OD1	A:ARG103:HH22—W:GLN61:O
A:ARG114:HH21—W:ASN54:OD1	A:ARG103:HH22—W:ILE62:O
A:HIS131:HD1—W:MET35:O	A:ARG107:HE—W:ASN54:OD1
A:GLN503:HE21—W:TYR33:O	A:ARG107:HH11—W:ARG57:O
W:ARG23:HH11—A:TYR230:OH	A:ARG107:HH12—W:GLN61:OE1
W:ARG23:HH21—A:TYR230:OH	A:ARG107:HH21—W:ASN54:O
W:ARG27:HE—A:GLU238:OE1	A:ARG107:HH22—W:ASN54:OD1
W:ARG28:HH11—A:GLN128:OE1	A:HIS131:HD1—W:MET35:O
W:ARG28:HH21—A:GLN128:OE1	W:ARG23:HH12—A:TYR230:OH
W:TYR33:HH—A:GLU506:OE2	W:ARG28:HH11—A:GLN128:OE1
W:MET35:H—A:GLU506:OE2	W:ARG28:HH21—A:GLN128:OE1
W:LEU36:H—A:GLU506:OE2	W:ARG28:HH21—A:GLN128:O
W:ILE38:H—A:GLU506:OE1	W:SER31:HG—A:ASN130:O
W:HIS47:HD1—A:GLN20:OE1	W:MET35:H—A:GLU506:OE2
W:TRP48:HE1—A:GLY583:O	W:LEU36:H—A:GLU506:OE2
W:ASN54:HD22—A:GLU111:OE2	W:ILE38:H—A:GLU506:OE1
W:ARG55:HE—A:GLU582:OE2	W:TRP48:HE1—A:GLY583:O
W:ARG55:HE—A:TYR584:OH	W:ASN54:HD21—A:GLU111:OE2
W:ARG55:HH21—A:GLU582:O	W:ASN54:HD22—A:GLU111:OE1
W:ARG57:HE—A:GLU111:OE1	W:ARG55:HE—A:GLU582:OE2
W:ARG57:HH11—A:ARG107:O	W:ARG55:HE—A:TYR584:OH
W:GLN61:HE22—A:ARG103:O	W:ARG55:HH11—A:GLU582:O
A:ARG13:CD—W:THR42:OG1	W:ARG55:HH21—A:GLU582:O
A:ARG103:CD—W:GLN61:O	W:GLN61:HE22—A:ARG103:O
W:GLY46:CA—A:GLN17:OE1	A:GLN503:CA—W:TYR33:O
W:ARG55:CD—A:GLU681:OE1	W:ARG23:CD—A:TYR230:OH
W:ARG59:CD—A:GLU681:OE2	W:GLY46:CA—A:GLN17:OE1
A:GLN20:HE22—W:HIS47	W:ARG59:CD—A:GLU681:OE2
	A:ARG245:NH2—W:TYR33

It should be noted that while the STAT3 structures between I-TASSER- and AlphaFold2-generated ones had a close RMSD value of 0.665 over 501 atoms upon superimposition, the GRIM-19 poses were found to be different between these two complexes. This might be due to the structural shifts occurring due to the displaced NTDs and the presence of C-terminal disordered regions in these two structures. However, it is a notable and an interesting observation that GRIM-19 residues in hydrogen bonding interactions are common and consistent across all the models studied. This consistency may indicate that these common GRIM-19 residues are absolutely required for the association with STAT3 molecule.

#### Molecular dynamics simulations

**Figure 4:**
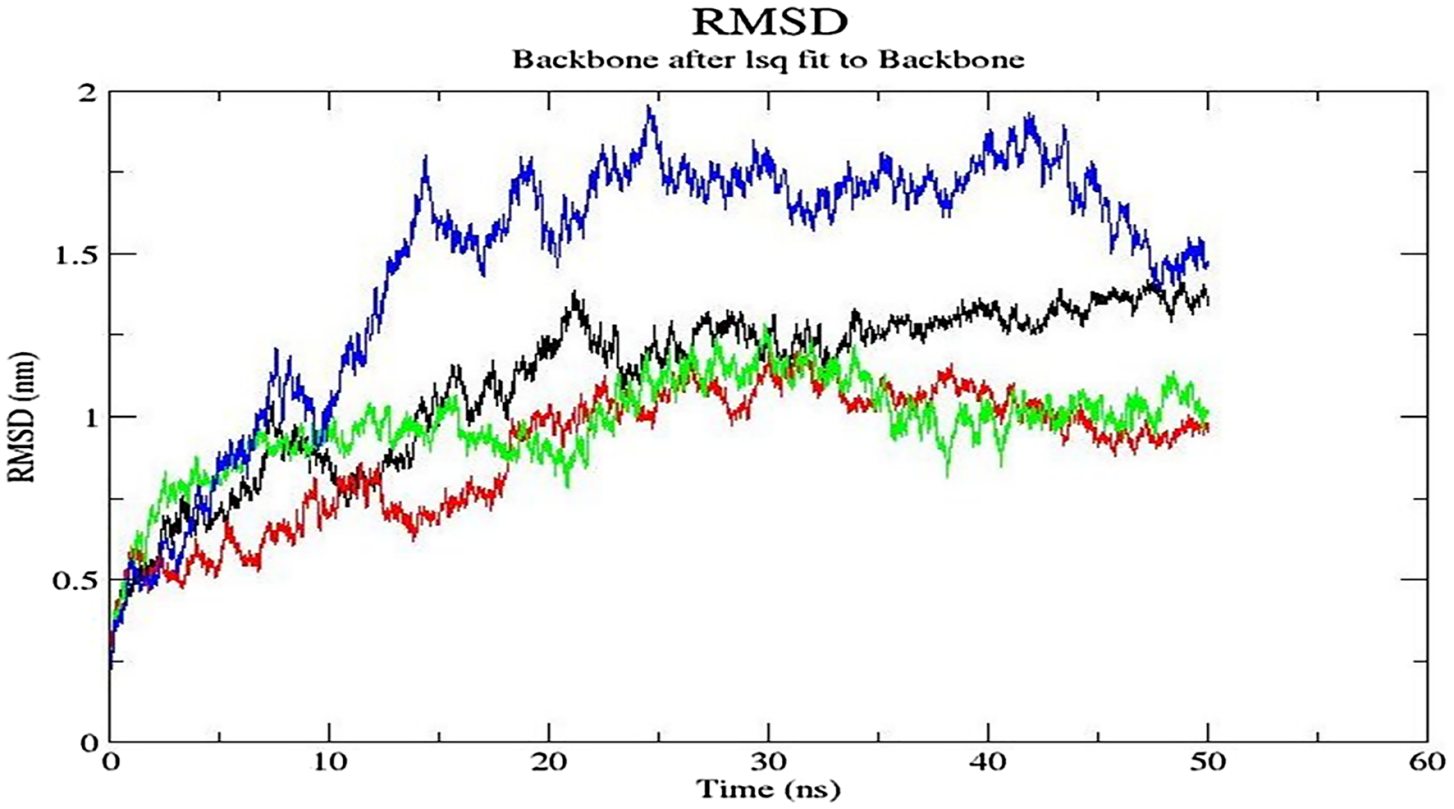
RMSD of the backbone atoms of the final structure relative to energy minimized structure vs. time plot for 50 ns simulation, for unphosphorylated and phosphorylated I-TASSER-generated STAT3-GRIM-19 complex (in black and red lines, respectively); and for unphosphorylated and phosphorylated AlphaFold2-generated STAT3-GRIM-19 complex (in green and blue lines, respectively).

Molecular dynamics studies were carried out using GROMACS version 2021 to assess the conformational stability of the four docked complexes: two complexes of GRIM-19 with unphosphorylated and phosphorylated I-TASSER-generated STAT3 and two complexes of GRIM-19 with unphosphorylated and phosphorylated AlphaFold2-generated STAT3. The RMSD values between the backbone atoms of the final structure relative to energy minimized structure are plotted as a function of time and shown in Fig. 4. We found that starting from 0.5 nm, the RMSD value hovered between 1 and 1.5 nm across the full 50 ns time period showing a stable plateau and therefore, a high stability of complexes. The phosphorylated AlphaFold2-generated STAT3-GRIM19 complex showed a slightly higher RMSD between 1.5 and 2 nm, but still plateaued at around the same time as other complexes. This slightly higher RMSD value in phosphorylated AlphaFold2-generated STAT3-GRIM19 complex may be due to flexible N- and C-terminal ends in GRIM-19 and flexible C-terminal end, which has high pLDDT score in AlphaFold2-generated STAT3 structure. While such flexible ends are present in all structures, the S727 phosphorylation in AlphaFold2-generated STAT3 at its C-terminal end may tend to slightly destabilize the complex conformation, while the same phosphorylation in I-TASSER-generated structure does not, indicating that the structure derived from homology-modeling may be more stable. Further, as we are observing the conformational effects of phosphorylation in a C-terminal disordered region, which is not expected to result in a drastic structural change as opposed to any phosphorylation event in the main folded part of a structure, this may also mean that, as of now, the homology-based modeling (e.g., using I-TASSER) may be a better computational approach than *ab-initio* modeling or those based on artificial intelligence (e.g., using AlphaFold2).

#### Venn diagram analysis

Despite the different binding site specificities observed, there were some common interaction patterns across multiple complexes. Common GRIM-19 residues in hydrogen-bonding across unphosphorylated and phosphorylated I-TASSER- and AlphaFold2-generated STAT3 were: Arg27, Tyr33, Arg55, Arg57, Gln61, Glu66 and Arg68 (supplementary Table S2). I-TASSER-generated structure was less sensitive to structural inducements while in a complex. GRIM-19 interactions were preserved. However, there were no common STAT3 residues across the I-TASSER-generated unphosphorylated and phosphorylated structures interacting with GRIM-19 while there were a total of 17 common interactions with GRIM-19 between unphosphorylated and phosphorylated AlphaFold2-generated STAT3 structures. This shows that Ser727 phosphorylation had little effect on these latter interactions, and its phosphorylation on the TAD C-terminal disordered region does not relay any conformational effect to the whole structure, so as to be able to enhance GRIM-19 association. There is a lack of enough information in the literature on the effect of S727 phosphorylation on STAT3 protein–protein interaction enhancement (16).

### C-terminal disordered region of GRIM-19

A loss of transmembrane potential in mitochondria was observed upon deletion of GRIM-19 residues 70–80, 90–100, and 70–144 (17). This shows that C-terminal region, even if it exists in a disordered state, is required for GRIM-19 activity. As we had taken full GRIM-19 structure in our docking studies mentioned above, in order to check out the interactions and poses of C-terminal domain-truncated GRIM-19 with I-TASSER-generated STAT3, we excluded these disordered regions at both the N- and C-terminal ends of GRIM-19. This is so because ClusPro2 is not very efficient at predicting complexes with disordered structures. It is a notable observation that the analyses revealed no significant major differences either way (supplementary Table S3), whether we took full-length GRIM-19 or GRIM-19 with truncated disordered regions for docking and binding analyses. This again proves the consistency of key important GRIM-19 residues in binding to STAT3, as well as confidence in ClusPro2 predictions with full-length GRIM-19.

### CC-DBD-LD and TAD domains of STAT3 in interactions with GRIM-19

Through our comprehensive blind docking approach, we identified NTDs of STAT3 as the major interacting sites of GRIM-19. However, as has been mentioned above, few studies (11, 12) did not detect the association of GRIM-19 with NTD and SH2 domains of STAT3 in their GST pull-down and immunoprecipitation experiments, respectively, as has been noted above. They observed CC-DBD-LD and TAD domains, respectively, in association with NTD of GRIM-19. The former study utilized murine constructs as well as overexpression of STAT3 and GRIM-19 in COS-1 cells. The caveat here is that COS-1 cells contain endogenous STAT3 as well, which may interfere with the effects of STAT3 overexpression. The latter study utilized STAT3-/- cells and found TAD domain as the major interactor site, in contrast to the former study. Hence, there is still a lack of clarity on the exact domains/binding sites of STAT3. Our comprehensive blind docking approach identified NTD of STAT3 as major interacting site. This occurred even when, besides blind docking, in another study, we specified attraction residues of just these CC-DBD-LD domains with a full-length STAT3 as input to ClusPro2, the NTD and SH2 domains of STAT3 again came in close contact with the NTD of GRIM-19. This observation leads to the insight that among several domains identified as possible binding sites, the NTD of STAT3 is the strongest contender based on fundamental principles of biophysical and biochemical processes.

### Dimeric STAT3 and GRIM-19 interactions in the cytoplasm

While our studies used a monomeric form of STAT3α to look into the binding sites analyses and generate a list of residues specific for interactions, we were also interested in studies on the dimeric form of STAT3α, especially because dimer structure is the predominant biologically active form in the cytoplasm. GRIM-19 is also found localized in the cytoplasm as noted above, hence, there is a distinct possibility that through co-localization, GRIM-19 may also interact with a dimeric form of STAT3. We wanted to perform comparison studies of monomeric and dimeric STAT3 to reflect on their abilities to bind GRIM-19.

As much as Y705 phosphorylation is required to induce dimer formation in active STAT3, the ability of latent, unphosphorylated STAT3 proteins to form dimers has been observed in experiments (17, 18). The N-terminal domain (1–125) was required for the formation of unphosphorylated dimers but not for tyrosine-phosphorylated dimers (18). Moreover, human STAT3β construct has been shown to form parallel homo-dimers (20), however, there is a truncation of N- and C-terminal ends in this structure where only residues 136–716 were taken to generate the dimer structure. The sequence for this expression construct is that of the mouse STAT3β, while our molecule of interest is human STAT3α. A full-length structure, rather than truncated parts, can affect the ability to form homodimers, and whether these are in a parallel or an anti-parallel orientation.

Y705- and S727-phosphorylated STAT3 has been widely studied as a dimeric molecule. In contrast, unphosphorylated STAT3 in a dimeric form is less studied and therefore, we focussed our attention towards a detailed understanding of its structural basis of action as a dimer. Further, since we have used the NTD-included structure and NTD is not required for the formation of tyrosine-phosphorylated dimers (17), we did not pursue the dimeric form of phosphorylated STAT3 for our studies.

#### Dimer structure generation

**Figure 5:**
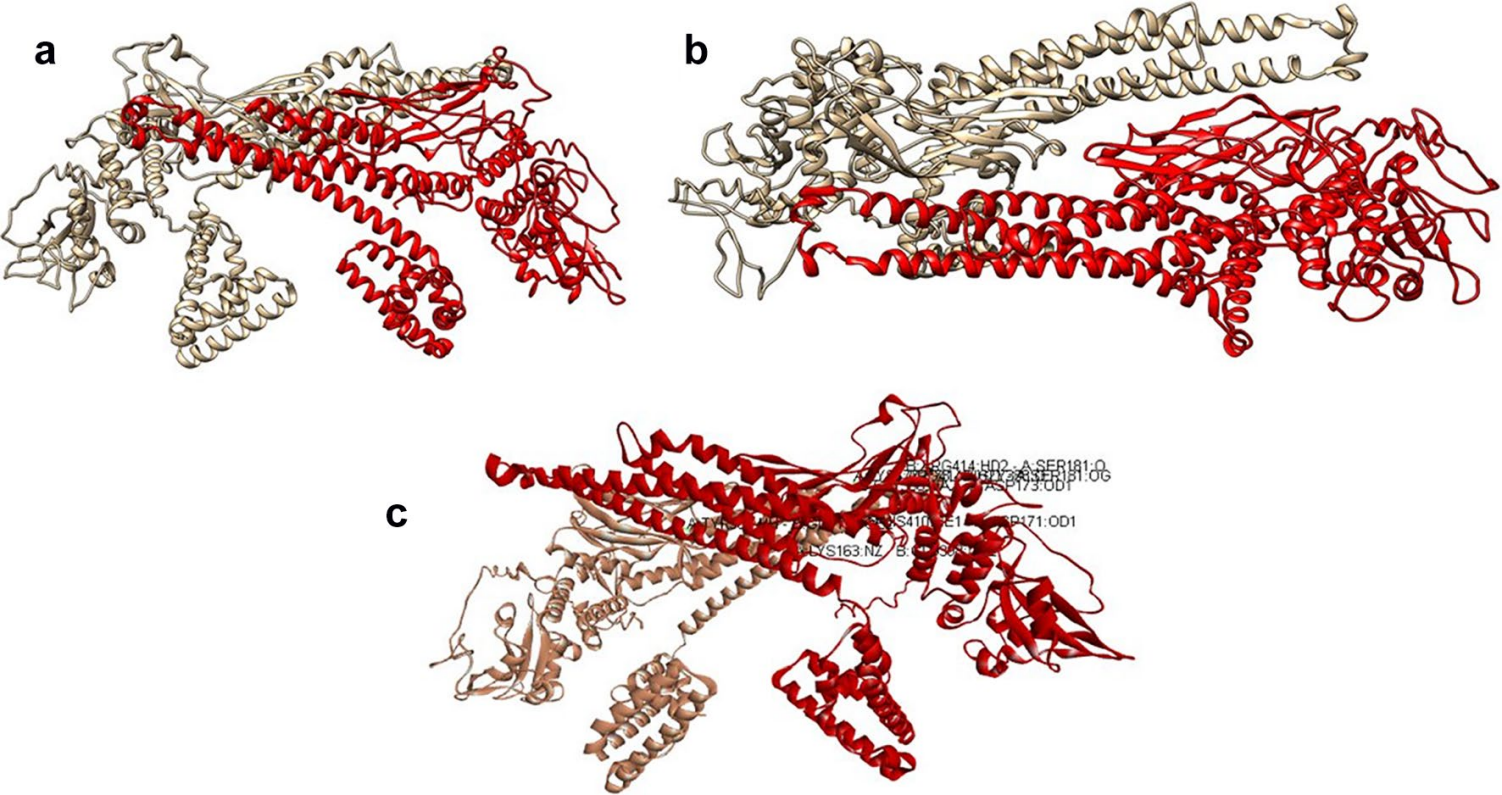
Dimeric structure of STAT3α (one monomer is in gold colour and the other monomer is in red colour) generated by Chimera MatchMaker using dimeric STAT1 (PDB ID 1YVL) as a template. (**a**) Side view, NTDs and DBDs face each other, (**b**) Top view, coiled coil domains in an antiparallel arrangement. (**c**) Hydrogen bond and electrostatic interactions between monomers at one end of STAT3α dimer identified using BIOVIA Discovery Studio.

Our full-length I-TASSER STAT3α structure is based on full-length STAT1 (PDB ID 1YVL) as a template; this template is present in an anti-parallel dimeric form in the crystallographic PDB coordinate file. We used this monomeric structure of unphosphorylated STAT3α to generate a biologically-relevant dimer on the basis of this same template using Chimera MatchMaker program. The dimer so obtained was in an anti-parallel orientation of two monomers (Fig. 5a). AlphaFold2-derived structure does not have a crystallographic template on which to model a biologically-relevant dimer, and so was modeled using the multimer mode in ClusPro2 to generate a dimeric structure. It was heartening to note that, indeed, all the top 10 dimeric molecules of AlphaFold2-generated structures (Supplementary zip file S1) were in an anti-parallel orientation similar to our dimeric structure derived from I-TASSER-generated monomer. This is in direct contrast to the studies using FRET which proposed parallel orientation of latent unphosphorylated STAT3 dimers; however, the identity of species (human or mouse) and the isoform (STAT3α or STAT3β), remains unknown (21).

In an anti-parallel dimer organization, the NTDs and DNA-binding domains of the two monomers face each other, and CCs and DBDs are more in contact than the rest of the structure. The coiled coil domains between NTDs and DBDs are arranged in a reverse fashion, sandwiching the DBDs between their long alpha-helical structures (Fig. 5b). The two SH2 domains, pY regions and C-terminal TADs are located at the farthest ends of the dimer. In this arrangement, most of the intermolecular interactions are likely to occur between DBDs and CC domains of each monomer, while the C-terminal region starting from SH2 domain is entirely devoid of any interactions with the opposite domains. This is shown by the hydrogen-bonded and electrostatic interactions between the two monomers with a total of 7 such interactions at one end as follows: Asp171-His410, Lys177-Gly388, Tyr360-Glu159, Arg414-Ser181 (two hydrogen bonds), Gly388-Asp173, Lys163-Glu398 and reciprocal interactions may occur at the other end (Fig. 5c).

**Figure 6:**
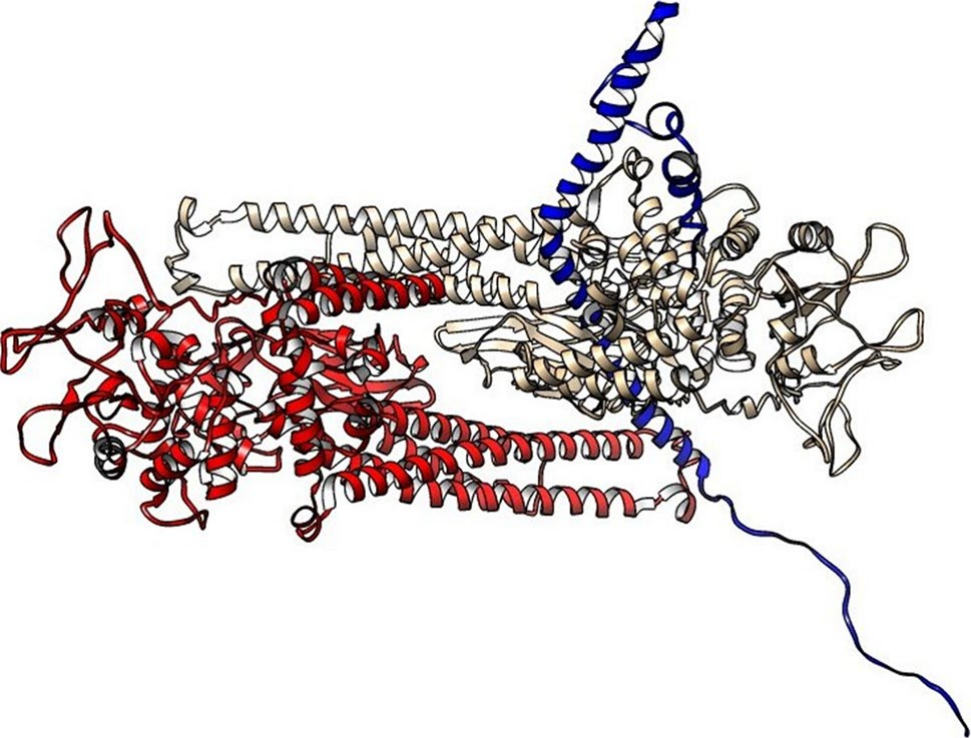
STAT3 dimers (monomers in gold and red colors) in complex with one molecule of GRIM-19 (in blue color).

As more confidence is gathered whenever an experimental template structure is available, we proceeded with this I-TASSER-derived dimeric structure of STAT3α to dock with GRIM-19. Dimeric STAT3α was energy minimized using DeepView and then fed into ClusPro2. The first generated hit according to the ClusPro2 cluster size was retrieved for further analyses on intermolecular interactions. GRIM-19 orientation and interactions with respect to STAT3α dimeric structure was more or less similar to the monomeric STAT3-GRIM-19 complex (Fig. 6, Supplementary Table S1). Tyr45, Arg55, Arg58 and Glu66 of GRIM-19 residues in hydrogen bond interactions with STAT3 residues overlapped between the monomeric and dimeric STAT3 structures.

### GRIM-19 protein: highly flexible, yet few arginines are constant in hydrogen-bond interactions across multiple sites of STAT3

The fully α-helical structure of GRIM-19 coupled with the presence of disordered regions at both ends lends it a greater degree of flexibility in terms of rotational and translational motions. FFT-based rigid docking implemented in ClusPro2 algorithm allows the rotation of ligand initially with 70,000 rotations narrowing down to clusters with 1000 rotation/translation combinations. Attempting to find the minimum energy conformations based on atomic interactions with STAT3, this allows for the difference in orientation and poses of GRIM-19 across the larger full-length STAT3 protein and its separated domains. The difference between the binding poses of GRIM-19, but not the interacting residues, across the longer full-length structure and shorter domains of STAT3 is also due to the size as well as differing atomic representations of the structures, and the ensuing nearest neighbour interactions.

Yet, as seen from the results above, in all of these binding modes which flexible GRIM-19 throws at us, is a conserved feature of Arginines (Arg57, Arg58 and Arg68) in hydrogen bonds across multiple STAT3 residues. We are yet to discern the exact STAT3 residues involved, since our first hit complex structures (both I-TASSER-derived and AlphaFold2-derived) all display differential interactions with the same arginines of GRIM-19. These arginines consistently found across multiple complexes studied in this paper provide a unique and crucial lead to the real nature of interactions occurring between these two important molecules. The electrostatic nature of interactions with GRIM-19 arginines is bound to play a larger role in the mitochondrial transmembrane potential, percolating down to influence myriad signal transduction pathways.

### STAT3-GRIM-19 mutations

#### Unphosphorylated and phosphorylated Ser727 of STAT3 in interactions with GRIM-19 and Ser727 mutations

Ser727 residue in TAD is well-known to be a critical residue required for the transactivation activity of STAT3. This amino acid residue is heavily invested in the activation of STAT3 through its phosphorylation effect. Its specific interactions with GRIM-19 on a structural basis, are as yet, unknown. Zhang et al*.* (11) found S727 to be required for GRIM-19 binding. Findings from their transfection and immunoprecipitation studies on select two mutations showed that S727A abolished 95% of GRIM-19 binding activity and S727E had no effect on this binding. They concluded from their S727E analysis that a negative charge is favorable at this position for GRIM-19 binding. Therefore, we were interested in studying the impact of S727 mutations on the STAT3-GRIM-19 binding affinity and stability on a structural basis.

Since only two mutations were studied above in the wet lab experiments, we proceeded to look at the effects of all of the 19 substitution mutations of S727 residue on the binding affinity of these proteins using tools which calculate changes in free energy upon mutation. There were two problems, though. S727 is located on STAT3 TAD which is a disordered region. ClusPro2 is not able to handle disordered regions well. Moreover, in all our top-most complexes studied, S727 was not found in direct hydrogen bonding, electrostatic and hydrophobic interactions with GRIM-19 in our full-length-docked structures.

The first problem of disordered region was solved when we found that GRIM-19 binds in the same orientation with or without its N- and C-terminal disordered regions. Hence, a degree of certainty is present. For the second problem, we scouted through all the top 10 complexes and found S727 in direct hydrogen bonding interactions within these top 10 complexes generated by ClusPro2. We found that such interactions were present in the 6th ClusPro2 complex model with I-TASSER-generated phosphorylated STAT3 and in 7th and 4th models with AlphaFold2-generated unphosphorylated and phosphorylated STAT3, respectively (Supplementary Table S4). We did not observe any Ser727 in hydrogen bonds in I-TASSER-generated unphosphorylated STAT3.

Initially, FoldX, a widely used tool to estimate the stability effects consequent upon mutation, was used (22). It calculates ΔΔG in kcal/mol, which is the difference in free energy between a wild-type and its mutant residue. ΔΔG > 0 kcal/mol is used as a threshold parameter for destabilisation of the structure, while the ΔΔG value < 0 kcal/mol is required to stabilise it. A mutation is considered significant if ΔΔG is > 1 kcal/mol.

The strength of the protein–protein interactions is determined by the binding affinity between two proteins, e.g., protein A and protein B. It is usually calculated by the following formula: dG_binding = dG_energy(AB complex)–dG_energy(A) – dG_energy(B). For stability studies, dG_stability is simply the value obtained by subtracting G_folded with G_unfolded, for a given protein going from an unfolded to a folded form, or perhaps from its native state to a more folded state residing in a complex owing to the conformational changes taking place. It should be noted that the term ‘stability’ here refers to binding stability and not necessarily conformational stability. The binding free energy change, ΔΔG_bind_, is thereafter, simply the difference between the binding energies of mutant and wild type complex.

For the binding affinities measurements, we used SSIPe tool (23), which scans the protein–protein interface to calculate the binding affinity changes (ΔΔG_bind_) of protein–protein interactions (PPIs) upon mutations. It combines weighted (to fit in experimental values) change in free energy calculated using sequence- and structure-based interface profiles with that of physics-based energy functions to obtain the final change in free energy score. We further wanted to study the effect of mutations of unphosphorylated and phosphorylated serine727 on the binding affinity of these complexes.

In an attempt to gain a consensus estimate, we also used coarse-grained approach in BeAtMuSiC (24) and machine-learning-based tool mCSM (25) to produce ΔΔG for binding affinity measurements. We observed that SSIPe, BeAtMuSiC and mCSM were in sync with respect to this conclusion: S727A mutation decreased the binding affinity, same as shown in the experiments (Supplementary Table S5). However, in the S727E case which was found to be neutral in the experiments, all these tools predicted the same behaviour of decreased binding affinity. BeAtMuSiC predicted that the mutation of S727 to D, the amino acid which belongs to same physico-chemical group as E, increases the binding affinity. Intuitively speaking, when physicochemical properties of serine, glutamic acid and aspartic acid are taken into account, serine being an uncharged amino acid and glutamic acid as well as aspartic acid being a negatively charged amino acid, these can have widely different mutational effects even though these may be semi-conserved due to their polarity. It is therefore, interesting to observe a neutral behaviour of this mutation occurring in the complex with GRIM-19. Such counter-intuitive neutral behaviour has also been observed in small-to-large mutation changes N → R (23). Mutation to all other amino acids also had the same effect of decreased binding affinity, with the most decrease in binding affinity accorded by mutation to proline. FoldX was not able to validate these experimental data in terms of stability, most probably due to the fact that this region is disordered, while FoldX is developed to predict the change in fold stability. The only residue mutation common with the above-mentioned tools was S727 mutation to proline resulting in destabilization. It should be noted that while FoldX estimates stability change upon mutation, SSIPe, BeAtMuSiC and mCSM all estimate binding affinity change of a complex upon mutation.

#### Mutations in interfacial residues and hotspots in STAT3-GRIM-19 complexes

Substitution mutations can affect a protein’s activity, its localization and interactions with other macromolecules. Apart from an effect on protein folding, such mutations can also stabilize or destabilize a protein–protein complex, existing either as a transient or a permanent complex. As STAT3 transcription factor is a multi-domain protein, mutations can also affect the domain-domain interactions resulting in the inhibition/activation of downstream signaling pathways.

We wanted to further observe unphosphorylated and phosphorylated STAT3-GRIM-19 binding through mutation analysis and resultant binding energy changes. Interrogation of specific or hotspot residues highly involved in the complex formation would provide us with a degree of revelation on those mutations which can modulate, increase or disrupt the binding affinity. As GRIM-19 is a known inhibitor of STAT3, and acts upon binding to it, understanding the mutation pattern of interfacial residues will help us in identifying those residues that are critically required to increase the binding affinity. Further, STAT3 NTD is predicted to be bound by GRIM-19 NTD in our studies, hence, most of the mutations occurring in the NTD region are likely to destabilize the complex formation.

From our S727 mutation analyses, which was guided by prior experimental data, we found SSIPe to be a good predictor of binding affinity and therefore, it was incorporated in our interfacial residues mutation analysis. Indeed, SSIPe is specifically developed to analyze interfacial mutations. This profile-based method is found to significantly outperform physics-based methods such as FoldX and methods based on a set of statistical potentials for coarse-grained representation of protein structures such as BeAtMuSiC in correctly predicting binding affinity change upon mutation (23). Moreover, as noted above, FoldX is optimized to predict fold stability and not binding affinity.

#### Binding affinity changes upon mutation

From I-TASSER-generated unphosphorylated or phosphorylated STAT3 partner side, SSIPe identified Y79P mutation as the one with most unfavorable mutation, it decreased the binding affinity the most (Table 3 and supplementary Table S6). The common GRIM-19 residue mutation causing the most unfavorable effect in complex with both unphosphorylated and phosphorylated STAT3 was Y33P. An interesting observation was that the tyrosine residue mutations at positions 33 and 45 topped the charts in having an unfavorable effect. The most favorable mutations in unphosphorylated and phosphorylated STAT3 in common, that could increase the binding affinity were: Q32L, K517L, and in GRIM-19, it was R58W.

**Table 3:** Binding free energy change (ΔΔG) values upon mutation of select residues, of interface residues involved in hydrogen bonding upon mutation to all other amino acids using SSIPe.

PP complex		Amino-acid mutations (ΔΔG in kcal/mol)										
		Y79P STAT3	Q32L STAT3	K517L STAT3	G583R STAT3	Q17L STAT3	E681W STAT3	Y33P GRIM-19	R58W GRIM-19	Q61L GRIM-19	R68P GRIM-19	R57M GRIM-19
I-TASSER	U	3.407	− 1.188	− 1.083	N	N	N	4.321	− 1.391	− 0.718	2.058	− 0.306
	P	3.367	− 0.551	− 1.144	N	N	N	3.472	− 1.367	− 0.714	1.795	− 0.364
AlphaFold2	U	N	N	N	3.196	− 1.192	− 1.063	3.312	N	− 1.202	2.296	− 0.078
	P	N	N	N	3.081	− 1.321	− 1.537	3.527	N	− 0.991	2.276	− 0.948

Positive values indicate decrease in binding affinity.

*N* not found in hydrogen bonds calculated by Discovery Studio, *U* Unphosphorylated STAT3-GRIM-19, *P* Phosphorylated STAT3-GRIM-19.

Likewise, analyses using AlphaFold2-generated structures (Table 3) showed that the most unfavorable mutation from unphosphorylated or phosphorylated STAT3 side was G583R, and from GRIM-19 side, it was the Y33P mutation, same as that observed in I-TASSER-generated STAT3 in complex with GRIM-19. The most favorable mutations in unphosphorylated and phosphorylated STAT3 were Q17L and E681W in common between the two. Q61L was the most favorable GRIM-19 mutation in unphosphorylated STAT3 complex and among the sixth most favorable mutation in complex with phosphorylated STAT3. Q61L was also the tenth amongst the most favorable mutations in GRIM-19 in complex with I-TASSER-generated STAT3 structures.

Among the common GRIM-19 arginine residues identified above in Venn diagram, several mutations led to decreased binding affinity with R68P topping the charts among arginines. However, a few arginine mutations such as R57M were observed to increase the binding affinities in all the complexes.

From the above analyses, it was obvious that while the mutations causing a favorable or an unfavorable effect were more or less the same in the case of GRIM-19, in the case of unphosphorylated and phosphorylated STAT3, these mutations were varied. There was a common pattern as these mutations were mostly located in the NTD region as well as the LD-SH2 region. The secondary structural elements involved in the interactions and mutation effect comprised of the helices, loops and unstructured regions of STAT3 and the helices of GRIM-19. Both the AlphaFold2-generated and I-TASSER-generated STAT3 structures in complex with GRIM-19 were observed to be most likely affected by Y33P mutation in GRIM-19.

Between unphosphorylated and phosphorylated STAT3 complexes, the line graphs showed major ΔΔG differences in Q32 and Q128 residue mutations in STAT3 in I-TASSER and AlphaFold2-generated structures, respectively (supplementary Fig. S1a, S1b, S1c, S1d). From the GRIM-19 perspective, the residues with most differences after mutations were R58 and Y33 (I-TASSER) and R57 and G46 (AlphaFold2). In AlphaFold2-generated STAT3, the GRIM-19 residue Y33 mutations had no notable difference between unphosphorylated and phosphorylated STAT3 complexes, while in I-TASSER-generated case, these displayed major differences in ΔΔG values. Mutations of the arginine residues at positions 57 and 58 of GRIM-19 had a major difference in the binding affinities in both these cases, again implicating arginines R57 and R58 as one of the major determinants of interactions. These residues may be taken as ‘hotspot’ residues along with the above-mentioned residues, the ΔΔG values calculated upon mutations of these point to a considerable impact on the binding affinity.

The negative values in SSIPe results indicate increased binding affinity with the strongest binding denoted by a mutation with ΔΔG ≤ − 1.5 kcal mol^-1^ while the least binding affinity is denoted by a mutation with ΔΔG ≥ 1.5 kcal mol^-1^. We averaged the positive and negative values separately to uncover the major contributions of any one partner towards the change in binding affinities. Analyses of these values (Table 4) show that in all the cases, in comparison to unphosphorylated and phosphorylated STAT3, GRIM-19 mutations displayed a larger average value, both in the increase or in the decrease of binding affinity. This implies that GRIM-19 has a larger role to play in the complex formation, and is more involved in the binding activity than is STAT3. This may also explain its specificity, as has been shown in the experiments where GRIM-19 does not bind to STAT1 or STAT5, but is highly specific to STAT3 (11). In the STAT3-GRIM-19 complex formation and interactions, GRIM-19 always has the upper hand whether STAT3 is phosphorylated or not.

**Table 4:** Average of all positive and negative binding free energy change (ΔΔG) values of interface residues involved in hydrogen bonding upon mutation to all other amino acids using SSIPe.

PP complex		A chain (STAT3)		W chain (GRIM-19)	
		Average of all positive ΔΔG values (kcal/mol)	Average of all negative ΔΔG values (kcal/mol)	Average of all positive ΔΔG values (kcal/mol)	Average of all negative ΔΔG values (kcal/mol)
I-TASSER	Unphosphorylated STAT3-GRIM-19	0.8717	− 0.3292	1.1061	− 0.4340
	Phosphorylated STAT3-GRIM-19	0.8620	− 0.3800	1.1293	− 0.3956
AlphaFold2	Unphosphorylated STAT3-GRIM-19	0.96973	− 0.3934	1.1956	− 0.3028
	Phosphorylated STAT3-GRIM-19	0.8400	− 0.5415	1.0492	− 0.4349

#### Stability studies upon mutation

After enlisting the key mutations that may increase or decrease the binding affinities, we further wanted to study the impact of these mutations on the stability of each monomer in the STAT3-GRIM-19 complex. Ideally speaking, if the binding affinity increases upon mutation, the stability of the constituent proteins should also increase, leading to the stabilization of the whole complex, thereby allowing proper functioning. Problems might occur if a mutation in a partner protein increases the binding affinity but decreases its stability. This would mean that the interactions with any partner protein harboring such a mutation may be more transient, mis-folding may occur, enough time would not lapse for interactions to take place properly, and hence activity or function may be compromised.

We used PositionScan of FoldX to generate mutants at the interfacial regions in our complexes and calculate the change in energies upon mutation. Initially, the interfacial residues were identified by using a python script interfaceResidues.py which can be downloaded by accessing PyMOL wiki interfaceResidues (https://pymolwiki.org/index.php/InterfaceResidues). These interfacial residues were then used in the input files for running FoldX, using the command PositionScan. Using PositionScan, we mutated all the input interfacial residues to the 20 naturally occurring amino acids and calculated the change in energy using ΔΔG = ΔGmut-ΔGwt. If the change in energy value is positive, it is indicative of destabilization upon mutation, and if it is negative, it is suggestive of mutations that can stabilize the partner proteins and thereby, the complex.

From our observations, for selected residues, we found that binding affinities calculated by SSIPe tallied with the stability of the monomers calculated by FoldX. All the mutations under consideration which were predicted to increase or decrease the binding affinities were observed to stabilize or destabilize the constituent proteins (Table 5 and also Supplementary Tables S7, S8, S9, S10). GRIM-19 Y33P and Q61L mutations in I-TASSER-generated STAT3 complex were contradictory in binding affinities and stability, although in AlphaFold2-generated STAT3 complex, these were totally in sync.

**Table 5:** Free energy change (ΔΔG) values upon mutation of select interfacial residues to analyse stability using FoldX.

PP complex		Amino-acid Mutations ( ΔΔG in kcal/mol)									R68P GRIM-19	R57M GRIM-19
		Y79P STAT3	Q32L STAT3	K517L STAT3	G583R STAT3	Q17L STAT3	E681W STAT3	Y33P GRIM-19	R58W GRIM-19	Q61L GRIM-19		
I-TASSER	U	2.90692	− 2.24753	− 2.19516	N	N	N	− **2.2876**	− 1.49793	**0.553864**	3.36941	− 1.4243
	P	2.76345	− 1.81479	Not seen in interface calculated by FoldX	N	N	N	− **1.13605**	0.337178	**0.956434**	4.35564	− 0.644057
AlphaFold2	U	N	N	N	11.1799	− 1.21722	**0.0417314**	2.13039	N	− 0.48772	2.28606	− 1.84743
	P	N	N	N	9.34793	− 1.8187	Not seen in interface calculated by FoldX	2.44019	N	− 1.89046	3.22277	− 0.267861

Positive values indicate destabilization.

*N* not found in hydrogen bonds calculated by Discovery Studio, *U* Unphosphorylated STAT3-GRIM-19, *P* Phosphorylated STAT3-GRIM-19, values highlighted in bold are the ones which contradict binding affinity observations.

## Discussion

STAT3 transcription factor is a multi-domain protein, playing multi-faceted roles in biological processes such as cell proliferation, cell survival, cell differentiation and cytokine-induced signalling responses. It has been found to function as a context-dependent oncogene or tumor suppressor and therefore, is an attractive drug target. Being subsequently found to be bound by GRIM-19 which exerts a negative regulatory effect on STAT3, the exact bound region of STAT3 is, as yet, an unresolved matter. From our studies, we found that the NTD region of GRIM-19 binds most strongly to the NTD of STAT3, even though experimental evidences in direct contradiction of one another, point to the other domains of STAT3. Even as discrepancies exist with respect to the STAT3 domains, one particularly interesting observation was that in both these experimental as well as our blind computational studies, the NTD of GRIM-19 was found to bind most strongly. GRIM-19 NTD binding occurred regardless of whether GRIM-19 was in a complex with the full-length STAT3 or with its individual domains, and whether it was bound to monomeric or dimeric STAT3. The feature that only the NTD of GRIM-19 is involved in the interactions with multiple regions/domains of STAT3 is an interesting topic deserving of further investigations. Even though the NTD and SH2 regions of STAT3 are not seen in complexes in experiments with murine constructs, these are observed from our docking studies with human proteins, carried out without specifying any residue in the interface (blind docking) and therefore, cannot be discounted. In fact, the NTD of STAT3 is the strongest contender for the binding site with GRIM-19, as seen from the results observed above. Even when we specified attraction groups of CC-DBD-LD and TAD, GRIM-19 again bound to only the NTD of STAT3.

The NTD of STAT3 has been shown to be involved in nuclear translocation, cooperative binding to the DNA and protein–protein interactions (19). Several studies have pointed to the importance of NTD of STAT3 in its activity. For its protein–protein interaction activity, post-translational modifications of NTD, such as acetylation, are required. STAT3 NH_2_-terminal acetylation stabilizes the STAT3–p300 complex, and this stabilization is necessary for target gene transcription (26). Further, STAT3 acetylation is also required for NTD of STAT3 to interact with HDAC1 (27). BRD4 associates more tightly with mono-ubiquitinated STAT3 in its NTD region (28).

Protein–protein interfaces, when subjected to mutations, may increase or decrease the association of involved proteins. It is frequently observed that mutations in the core region alter the stability of a molecule while mutations at the surface which are frequently found in interfacial regions, at or near the hotspot region, may affect binding affinity. In an ideal environment, a mutation that increases or decreases binding affinity should also confer increased or decreased stability, respectively, and it is useful to pinpoint such mutations. Situations may occur when a mutation has a stabilizing effect on folding but destabilizing effect on binding, and in such a scenario, it is entirely plausible that there may be other factors coming into play, such as the natural selection acting at a local rather than a global level (29).

From our mutation analysis, incorporating the change in binding free energy as well as the change in stability upon mutation, interfacial GRIM-19 residue mutations most involved in decreasing and increasing the binding affinity and stability were: Y33 and Q61 mutations, respectively. Mutations of the GRIM-19 residues R57 and R58, alongwith Y33, displayed notable differences in binding affinities when comparing the unphosphorylated and phosphorylated forms of STAT3 in the complexes. It may also be worth noting that the mutations which increase the binding affinity are less well studied than those which decrease the binding affinity in experiments, which may be a limitation in our analyses. With decreased binding affinity due to the introduction of mutations, a major consequence could be the disruption of protein–protein complex formation and resultant signalling. In contrast, mutations that increase the affinity may over-stabilize protein–protein complexes that are functional only when in a transient complex and may disrupt proteostasis (30, 31). This proteostasis disruption may further affect or modulate downstream signalling. In this manner, these changes in the magnitude of the energy due to the mutations in the STAT3-GRIM-19 complexes, may alter the strength of signalling as well as the respective activities.

Further, the mutations of amino acid residues from larger-to-smaller volume or from smaller-to-larger volume, may change the energy function drastically, all the more so in the latter case, which typically assumes the backbone of a protein to be fixed. Physicochemical changes in the residues residing in a PPI interface also play a major role (30). Intuitively, interfacial structure, conformation, and stability of STAT3-GRIM-19 complex can be strongly affected by volume as well as large physicochemical changes such as Y → P (larger-to-smaller) and G → R (smaller-to-larger) amino acid mutations, as noted above. These mutations are likely to have a major impact on the binding process of these two molecules. On the other hand, Q → L (medium → large) mutations may be typically favorable owing to lesser magnitude in the volume increase and may tend to increase the binding affinity. It is of note that Y → P and Q → L mutations change from polar to non-polar class, while G → R mutation is from non-polar to polar class of aminoacids. Therefore, while Q → L mutation can affect binding in terms of the change in its physicochemical criterion, when considered in terms of volume change, the effect is insignificant.

## Conclusions

Based on the near-accurate STAT3 structures, our work provides a novel perspective on the STAT3 sites bound by GRIM-19, as compared to that observed from the earlier experiments. These experimental observations are inconsistent among each other and so, clarity needs to be established. We found that NTD of GRIM-19 binds to and interacts with NTD of STAT3α most strongly, and key arginine residues of GRIM-19 are ubiquitous interacting residues across all of the studied complexes. This new perspective will pave a way forward in understanding how GRIM-19 binds specifically to STAT3, and how the introduction of mutations at the interface disrupting the hydrogen bonding interactions may lead to the development of effective protein therapeutics including GRIM-19, which is a known inhibitor of STAT3. Our study also delves into an understanding of the mechanism of action of GRIM-19. Further, analyses using mutagenesis experiments in human cell or tissue context will shed more light into the binding specificity, sensitivity and mechanisms of action.

## Materials and methods

### Sequence and structure retrieval

The full-length sequence of human STAT3α was downloaded from UniProt ID: P49763. Its full-length structure was downloaded from AlphaFold2 database hosted at EBI with same UniProt ID P40763 (https://AlphaFold2.ebi.ac.uk/, 32). The structure of human GRIM-19 was retrieved from PDB ID: 5XTD (W chain). Both the GRIM-19 and STAT3 structures were energy minimized before use.

### STAT3α structure modelling

To carry out comparison studies and to generate consensus results owing to varying levels of confidence, the 770 bp STAT3α sequence was subjected to homology modelling with I-TASSER (https://zhanglab.dcmb.med.umich.edu/I-TASSER/, 33) and SWISS-MODEL (https://swissmodel.expasy.org/, 34). I-TASSER is the number one server for protein structure prediction as identified by several CASP experiments. It first uses threading approach LOMETS to identify template structures from the PDB and then, on the basis of template with highest Z-score, constructs full-length atomic models. SWISS-MODEL works on the principle of target-template alignment and final structures are selected using statistical potentials of mean force scoring. If there are are no such candidates, then Monte Carlo approach is used to search for conformational space and then the structures are minimized using SCWRL4 energy function. Both I-TASSER and SWISS-MODEL identified human STAT1 (PDB ID: 1YVL) as a significant template. The first modelled hit was downloaded and energy minimized using Swiss PDB Viewer (https://spdbv.vital-it.ch/) to carry out further analyses. This unphosphorylated structure was also subjected to computational S727 phosphoryl group attachment using BIOVIA Discovery Studio Visualizer in order to generate a phosphorylated molecule. After the addition of phosphoryl group, it was again energy minimized to remove any steric clashes.

### Model quality assessments

We used Structure Analysis and Verification Server (SAVES, https://saves.mbi.ucla.edu/) to generate ERRAT and Verify3D assessments and PDBsum (https://www.ebi.ac.uk/thornton-srv/databases/cgi-bin/pdbsum/GetPage.pl?pdbcode=index.html) to generate PROCHECK results based on Ramachandran plots on model quality.

### STAT3α dimer generation

Chimera (https://www.cgl.ucsf.edu/chimera/) Matchmaker tool was used to generate STAT3α dimer structure based upon template STAT1 (PDB ID 1YVL). This template is present as a dimer in the PDB file, and so the monomeric STAT3α was superimposed upon the dimers to generate a biologically-relevant dimeric STAT3α structure.

### ClusPro2 protein–protein docking

ClusPro2 (https://ClusPro2.bu.edu/login.php, 35) is amongst the top-most protein–protein complex generation tool as verified by independent CAPRI assessments, and is widely used. It works on the principle of fast Fourier transform correlation, and then the outputs are filtered using a combination of desolvation and Coulombic electrostatic energies. Thereafter, these filtered structures are clustered to incorporate a representative structure nearest the global minimum, which is the final structure. Using the blind docking (default protocol) in ClusPro2, which is used without specifying any interface/interacting residues, we generated several complexes of STAT3α-GRIM-19 structure, using both I-TASSER- and AlphaFold2-generated STAT3α structures, as follows: unphosphorylated STAT3-GRIM-19, phosphorylated STAT3-GRIM-19, unphosphorylated STAT3α-CTD truncated region of GRIM-19, unphosphorylated CC-DBD-LD region of STAT3-GRIM-19 and unphosphorylated dimeric STAT3-GRIM-19. The smaller of the two proteins was input as ‘ligand’ and the larger one as ‘receptor’ during docking. Hydrogens were removed prior to docking since ClusPro2 adds polar hydrogens before docking during input structure preparation. From the results generated, mostly, the topmost hit generated on the basis of the biggest cluster size according to ClusPro2 was taken for further analyses. In order to generate a dimeric structure from AlphaFold2-predicted monomeric STAT3α, multimer docking mode of ClusPro2 was used.

### Visualization

Structural visualizations were done using BIOVIA Discovery Studio and UCSF Chimera tools. Intermolecular interactions were calculated using Discovery Studio.

### Molecular dynamics studies

The input files for molecular dynamics were generated using CHARMM-GUI (http://www.charmm-gui.org, 36) v3.5 with GROMACS-formatted CHARMM36 forcefield for both phosphorylated and unphosphorylated protein complexes and default parameters. These input files were subjected to 5000 steps of steepest descent minimization and 100 ps of equilibration as per CHARMM-GUI protocol. Verlet cut-off scheme for all minimization and equilibration steps was used. The input files so generated were subjected to 50 ns production run with GROMACS version 2021. A constant temperature of 303.15 K and constant pressure of 1 bar with integration time step of 2 fs was used. All bond lengths were constrained using LINCS algorithm. For long range electrostatic interactions, Particle-mesh Ewald (PME) algorithm was used (37) and short-range electrostatics and short-range van der Waals cutoffs were set at 1.2 nm.

### Mutation analyses: binding affinity and stability studies

The complexes obtained from ClusPro2 were used as an input to SSIPe (https://zhanglab.ccmb.med.umich.edu/SSIPe/, 23), BeAtMuSiC (http://babylone.ulb.ac.be/beatmusic/, 24) and mCSM (http://biosig.unimelb.edu.au/mcsm/, 25) to predict binding affinity changes upon mutation and FoldX to predict change in stability upon mutation.

SSIPe is specifically developed for a protein–protein interface analyses to calculate binding affinity changes (ΔΔGbind) upon mutations. It generates sequence and structural profiles from PDB and STRING databases and then combines the results with physical energy function, EvoEF. EvoEF takes into account the addition of energies arising from van der Waals, electrostatics, desolvation and hydrogen bonding interactions and then subtracts it with summed reference energy of the amino acids in the protein sequence as follows:

22 $${\text{E}}_{{{\text{EvoEF}}}} = {\text{ E}}_{{{\text{VDW}}}} + {\text{ E}}_{{{\text{ELEC}}}} + {\text{E}}_{{{\text{HB}}}} + {\text{ E}}_{{{\text{DESOLV}}}} - {\text{ E}}_{{{\text{REF}}}}$$

The final change in energy upon mutation is then calculated as

$$\Delta \Delta {\text{Gbind}} = \Delta {\text{Gbindmut}} - \Delta {\text{Gbindwt}}$$

where ΔGbind = binding energy, mut = mutant, wt = wild-type.

While BeAtMuSiC depends upon known statistical potentials from protein structures and takes into account the conformational rearrangements that can occur after the individual monomers bind, mCSM is a machine learning protocol that uses signatures which are patterns of distance between neighbouring atoms surrounding a protein residue. FoldX calculates stability change upon mutation, and uses empirical effective energy function (EEEF) to determine the free energy change upon mutation to represent stability.

Default parameters were used in all of these tools. The interface residues chosen were those in hydrogen bonding interactions across the complexes. These interface residues were then subjected to mutations to all other 19 naturally occurring amino acids. For FoldX4, the ClusPro2-minimized complexes were subjected to PositionScan to mutate the interface residues calculated by interfaceResidues.py PyMol script, and then the difference in energy upon mutation was calculated. The ΔΔG values so obtained from all of these tools were then ranked according to descending order of these values representing binding affinity and stability.

### Significance statement

STAT3 transcription factors are multi-domain proteins playing a crucial role in biological processes and depending upon the context, may act as an oncogene/tumor suppressor. GRIM-19 is a highly specific biological inhibitor, negatively regulating STAT3, and the structural basis of its action is as yet, unknown. Using atomistic modeling with I-TASSER and AlphaFold2 structures, our comprehensive studies underscore the importance of key GRIM-19 residues playing a role in STAT3 binding and stability through mutation studies, and provide novel insights into the involvement of NTDs of both these molecules.

## Supplementary Information

Supplementary Legends.

Supplementary Table 1.

Supplementary Table 2.

Supplementary Table 3.

Supplementary Table 4.

Supplementary Table 5.

Supplementary Table 6.

Supplementary Table 7.

Supplementary Table 8.

Supplementary Table 9.

Supplementary Table 10.

Supplementary Information.

Supplementary Figure S1.

Supplementary Figure S2.

Supplementary Figure S3.

Supplementary Figure S4.
