# Supplementary material for: Structural exploration with AlphaFold2-generated STAT3α structure reveals selective elements in STAT3α-GRIM-19 interactions involved in negative regulation
Source: Sci Rep. 2021 Nov 30;11:23145. doi: 10.1038/s41598-021-01436-7 (PMC8633360; doi:10.1038/s41598-021-01436-7)
Supplement: Supplementary file 1 — Supplementary Legends. [file 41598_2021_1436_MOESM1_ESM.docx]

**Supplementary Information:**

Supplementary Table S1 in supplementary materials: Model quality assessments.

Supplementary Table S2: Venn diagrams: Common GRIM-19 residues in hydrogen-bonding across unphosphorylated and phosphorylated iTASSER- and AlphaFold2-generated STAT3.

Supplementary Table S3: CTD-deleted GRIM-19 interactions with STAT3.

Supplementary Table S4: S727 in hydrogen bonds with GRIM-19.

Supplementary Table S5: SSIPe, BeAtMuSiC and mCSM results for S727 mutations.

Supplementary Table S6: SSIPe mutation data (ΔΔG values) for unphosphorylated and phosphorylated STAT3 complexes.

Supplementary Table S7:FoldX stability ΔΔG values upon mutation, AlphaFold-generated phosphorylated STAT3-GRIM-19, Chain A: STAT3, Chain W: GRIM-19.

Supplementary Table S8:FoldX stability ΔΔG values upon mutation, AlphaFold-generated unphosphorylated STAT3-GRIM-19, Chain A: STAT3, Chain W: GRIM-1.

Supplementary Table S9:FoldX stability ΔΔG values upon mutation, iTASSER-generated phosphorylated STAT3-GRIM-19, Chain A: STAT3, Chain W: GRIM-1.

Supplementary Table S10:FoldX stability ΔΔG values upon mutation, iTASSER-generated unphosphorylated STAT3-GRIM-19, Chain A: STAT3, Chain W: GRIM-1.

Supplementary zip file S1: AlphaFold2-generated STAT3 as a dimer.

Supplementary Fig. S1a, S1b, S1c, S1d:Line graphs showing major ΔΔG differences between unphosphorylated and phosphorylated STAT3 complexes.
