# Supplementary material for: Structural exploration with AlphaFold2-generated STAT3α structure reveals selective elements in STAT3α-GRIM-19 interactions involved in negative regulation
Source: Sci Rep. 2021 Nov 30;11:23145. doi: 10.1038/s41598-021-01436-7 (PMC8633360; doi:10.1038/s41598-021-01436-7)
Supplement: Supplementary file 2 — Supplementary Table 1. [file 41598_2021_1436_MOESM2_ESM.docx]

## Supplementary table S1

## PROCHECK statistics for DeepView-minimized iTASSER model

**1. Ramachandran Plot statistics**

**No. of**

**residues %-tage**

**------ ------**

Most favoured regions [A,B,L] 535 77.0%******

Additional allowed regions [a,b,l,p] 133 19.1%

Generously allowed regions [~a,~b,~l,~p] 20 2.9%

Disallowed regions [XX] 7 1.0%*****

---- ------

Non-glycine and non-proline residues 695 100.0%

End-residues (excl. Gly and Pro) 2

Glycine residues 39

Proline residues 34

----

Total number of residues 770

## PROCHECK statistics for DeepView-minimized AF model

## 1. Ramachandran Plot statistics

**No. of**

**residues %-tage**

**------ ------**

Most favoured regions [A,B,L] 629 90.5%

Additional allowed regions [a,b,l,p] 58 8.3%

Generously allowed regions [~a,~b,~l,~p] 6 0.9%

Disallowed regions [XX] 2 0.3%*****

---- ------

Non-glycine and non-proline residues 695 100.0%

End-residues (excl. Gly and Pro) 2

Glycine residues 39

Proline residues 34

----

Total number of residues 770

Based on an analysis of **118** structures of resolution of at least **2.0** Angstroms and R-factor no greater than **20.0** a good quality model would be expected to have over **90%** in the most favoured regions [A,B,L].
