# Supplementary figures and images for: Structural exploration with AlphaFold2-generated STAT3α structure reveals selective elements in STAT3α-GRIM-19 interactions involved in negative regulation
Source: Sci Rep. 2021 Nov 30;11:23145. doi: 10.1038/s41598-021-01436-7 (PMC8633360; doi:10.1038/s41598-021-01436-7)

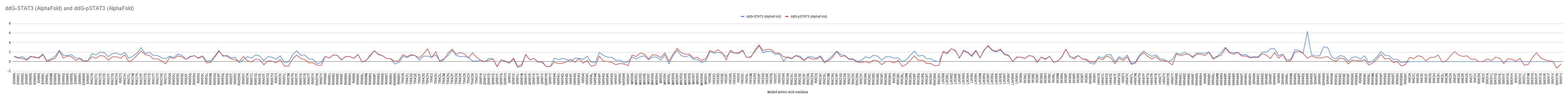

Supplement: Supplementary file 13 — Supplementary Figure S1. [file 41598_2021_1436_MOESM13_ESM.png]

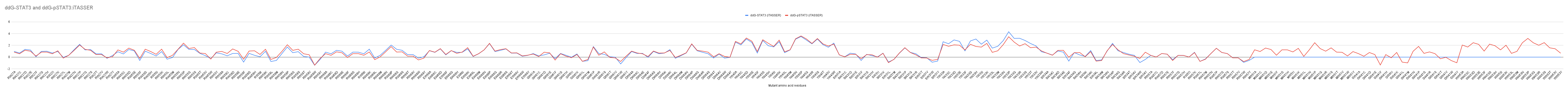

Supplement: Supplementary file 15 — Supplementary Figure S3. [file 41598_2021_1436_MOESM15_ESM.png]

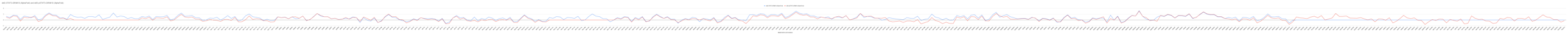

Supplement: Supplementary file 16 — Supplementary Figure S4. [file 41598_2021_1436_MOESM16_ESM.png]
